# BetaSearch: a new method for querying *β*-residue motifs

**DOI:** 10.1186/1756-0500-5-391

**Published:** 2012-07-30

**Authors:** Hui Kian Ho, Graeme Gange, Michael J Kuiper, Kotagiri Ramamohanarao

**Affiliations:** 1 Department of Computing and Information Systems, The University of Melbourne, Victoria, Australia; 2 National ICT Australia (NICTA), The University of Melbourne, Victoria, Australia; 3 Victorian Life Sciences Computation Initiative (VLSCI), The University of Melbourne, Victoria, Australia

## Abstract

**Background:**

Searching for structural motifs across known protein structures can be useful for identifying unrelated proteins with similar function and characterising secondary structures such as *β*-sheets. This is infeasible using conventional sequence alignment because linear protein sequences do not contain spatial information. *β*-residue motifs are *β*-sheet substructures that can be represented as graphs and queried using existing graph indexing methods, however, these approaches are designed for general graphs that do not incorporate the inherent structural constraints of *β*-sheets and require computationally-expensive filtering and verification procedures. 3D substructure search methods, on the other hand, allow *β*-residue motifs to be queried in a three-dimensional context but at significant computational costs.

**Findings:**

We developed a new method for querying *β*-residue motifs, called BetaSearch, which leverages the natural planar constraints of *β*-sheets by indexing them as 2D matrices, thus avoiding much of the computational complexities involved with structural and graph querying. BetaSearch exhibits faster filtering, verification, and overall query time than existing graph indexing approaches whilst producing comparable index sizes. Compared to 3D substructure search methods, BetaSearch achieves 33 and 240 times speedups over index-based and pairwise alignment-based approaches, respectively. Furthermore, we have presented case-studies to demonstrate its capability of motif matching in sequentially dissimilar proteins and described a method for using BetaSearch to predict *β*-strand pairing.

**Conclusions:**

We have demonstrated that BetaSearch is a fast method for querying substructure motifs. The improvements in speed over existing approaches make it useful for efficiently performing high-volume exploratory querying of possible protein substructural motifs or conformations. BetaSearch was used to identify a nearly identical *β*-residue motif between an entirely synthetic (Top7) and a naturally-occurring protein (Charcot-Leyden crystal protein), as well as identifying structural similarities between biotin-binding domains of avidin, streptavidin and the lipocalin gamma subunit of human C8.

## Background

**Figure 1:**
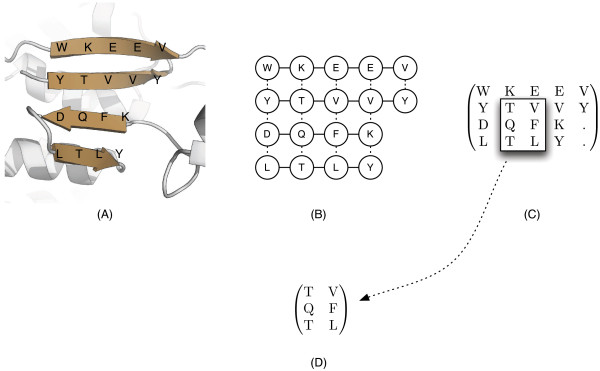
**An example *β*-sheet with its corresponding *β*-graph, *β*-matrix, and *β*-residue motif.** Here we show an example conversion of a *β*-sheet (**a**) to its graph representation (or “*β*-graph”) (**b**) and the *β*-matrix projection (**c**). A *β*-residue motif (**d**) is a sub-matrix of its source *β*-matrix, or a connected subgraph of its source *β*-graph. The source code to perform these conversions are available for download (see Availability).

The *β*-sheet is a common secondary structure element that plays important functional and structural roles in proteins, for example, the ligand-binding pockets of biotin-binding proteins and the structure of the commonly-occurring TIM-barrel fold [1]. These processes are often mediated by interactions between adjacent pairs of residues across *β*-strands. These include the disulphide, ionic, and hydrogen bonds; and hydrophobic packing interactions frequently involved in maintaining the structural stability of a protein or in enzymatic active sites [1]. The influence of pairwise interactions within *β*-sheets and their tertiary structures have been studied experimentally [2] and statistically [3,4], the results of which have been used to predict *β*-sheet topology [5-7] and tertiary structure [8]. These studies have provided insights into the folding mechanisms of *β*-sheets although it remains an open problem [4]. Examining interresidue interactions at the single pairwise level however, provides only a limited view of a larger interaction network within a *β*-sheet. We refer to these clusters of interacting residues as *βresidue motifs*, which are contiguous subsets of *β*-sheet residues connected by peptide and/or hydrogen bonds (as shown in Figure 1D). Unlike sequence motifs, *β*-residue motifs encode information about both the peptide and bridge-partners of each residue. For the purposes of this study, we consider *β*-residue motifs to be provisional, since they may also exist via a general conservation between homologs rather than as independent functional units, as is the case for motifs in the traditional sense [9].

Many characteristic *β*-residue motifs are observed in the Protein Data Bank (PDB) [10]. For example, the *β*-sheets in leucine-rich repeat (LRR) domains contain consecutive adjacent interstrand pairs of buried leucines sterically-packed alongside their bridge-partners, contributing to structural stability [11]. Other *β*-residue motifs appear as a combination of inter- and intrastrand residue neighbours, as is the case for the TCT motif of certain antifreeze proteins [12] and glutamic acid/lysine motifs [13]. The conserved biotin-binding site in streptavidin [PDB:1STP] contains a *β*-residue motif of five inward-directing residues of a *β*-barrel: S88, T90, W92, W108, and L110. Identification of these *β*-residue motifs can be used to search for other proteins with similar structural elements or function with low sequence identities.

Searching for structural motifs, *β*-residue or otherwise, in the PDB using linear approaches such as sequence alignment is a difficult, if not impossible task because interresidue interactions can occur across secondary structures that are sporadically located throughout a protein sequence. Furthermore, pairwise residue interactions are not accounted for in conventional multiple-sequence alignment tools such as BLAST [14] and CLUSTALW [15], given the one-dimensional nature of sequences.

The conventional approach to motif querying, involves the use of protein substructure search methods that structurally align the 3D atomic coordinates of a query with known protein structures. These methods provide a structural context to each query hit and generally produce approximate matches in the form of a ranked list of hits but may take hours to perform few queries due to their reliance on structural alignment algorithms [16].

Alternatively, protein structures can be represented as graphs and queried for motifs using graph indexing approaches [17]. Unlike 3D substructure searches, these methods perform exact matching by querying only the discrete edge, node, and label features of graphs rather than by 3D similarity between continuous coordinates. The query matching algorithms used by existing graph indexing methods are based on solutions to the subgraph isomorphism problem, described briefly as follows:

A *graphG*=(*V*,*E*) is defined by a set of vertices *v*∈*V*and a set of edges *e*∈*E*where each edge represents a connection between a pair of vertices $\left(v_{i},v_{j}\right)\in V$. A graph is *undirected* if its edges are unordered pairs and *directed* otherwise. The *degree* of a node is the number of edges it has to other nodes.

If *G*_1_and *G*_2_are graphs defined as $G_{1}=\left(V_{1},E_{1}\right)$ and $G_{2}=\left(V_{2},E_{2}\right)$, then *G*_1_is a *subgraph* of *G*_2_if $V_{1}\subseteq V_{2}\;\;\wedge \;\;E_{1}\subseteq \;E_{2}$. An *isomorphism* between *G*_1_and *G*_2_is a bijection *f*:*V*_1_→*V*_2_such that $\forall \left(v_{i},v_{j}\right)\in E_{1}\;\Leftrightarrow \;\left(f(v_{i}),f(v_{j})\right)\in E_{2}$.

A graph *G*_1_is *subgraph isomorphic* to *G*_2_if *G*_3_is a subgraph of *G*_2_and there exists an isomorphism between *G*_3_and *G*_1_.

Graph representations of proteins [18] and *β*-sheets [17] have been previously described in which nodes represent residues and edges represent inter-residue interactions such as peptide or hydrogen bonds. For simplicity, we define a *βgraph* to be a graph representation of a *β*-sheet in which each node is labelled with a residue name, solid edges represent peptide bonds, and dotted edges represent a bridge-partner relationship between adjacent interstrand residue pairs (Figure 1B). *β*-residue motifs are considered to be connected subgraphs of *β*-graphs whose nodes are labelled with amino acids.

The planarity of *β*-graphs allows for a compact two-dimensional representation. We define a *β*-matrix to be a projection (or “flattening”) of a *β*-graph onto a 2D matrix of amino acid characters. The residues in the same *β*-strand are located in the same row and residues connected by bridge edges lie in the same column (Figure 1C). *β*-residue motifs are then considered to be submatrices of *β*-matrices (Figure 1D).

Algorithms for detecting subgraph isomorphisms have been described for general graphs [19,20] that run in time factorial to the number of vertices in a query [21] and cannot be solved in polynomial time, as it is proven to be NP-complete for general graphs [22]. Naively performing a subgraph test on every graph in a database is therefore computationally-expensive [23]. Consequently, graph indexing methods were developed to simplify this problem. A potentially large number of non-matching graphs can be pruned from the search by using indexing techniques analogous to those in conventional search engines [24]. Graphs can be indexed by various features using disk- or memory-based indices. These approaches usually consist of three stages: 

1. **Index construction**: The features of each graph in a database are obtained, each representing a graph characteristic. A data structure, usually an inverted index, is then constructed in which each feature is associated with the set of their originating graphs.

2. **Filtering**: The features of the query graph are obtained. An initial set of coarse-grained candidates containing these features are retrieved from the index (or indices). It is possible these candidates do not contain any query matches.

3. **Verification**: Each candidate is checked for a subgraph that exactly matches the query. This is performed using a subgraph test in most methods.

Graph indexing methods are loosely classified into three categories: path-based, subgraph-based, and tree-based.

Path-based methods (GraphGrep [25], GraphFind [26], GraphGrepSX [23], and SING [27]) index graphs using paths as features. These methods construct an inverted index *I* that maps each path *p* to a set of their originating graphs

(1) I:p↦{G:p∈G}

where *p* is a sequence of connected vertices in a graph *G*

(2) $$\begin{array}{ll} p & =\left(v_{i},v_{i+1},\cdots \;,v_{i+k-1}\right)\;\\ \forall k & :1\leq k\leq l_{p}\; \end{array}$$

where *l*_*p*_ is the maximum path length. The filtering process returns the set of candidate graphs *C* containing all the paths of a query graph *Q*

(3) $$C=\underset{p\;\in \;\text{paths}(Q)}{⋂}I(p)$$

and verification of each candidate is performed using the VF2 algorithm [20].

Enumerating all paths up to and including length *l*_*p*_produces large feature sets and consequently, large indices. GraphFind avoids these problems by pruning redundant features using data mining techniques similar to those of gIndex [28]. GraphGrepSX exploits feature redundancy by implementing its index as a suffix tree where each string is a path sequence. The suffix tree was shown to be more space-efficient than the hash tables used by other path-based methods [23]. Our empirical results corroborate these findings (Table 1). SING uses a second filtering stage that prunes candidates by using path locality information. For example, a path *p* in a candidate must be surrounded by the same paths as in the query. This improvement in filtering comes at the cost of maintaining an auxiliary hash table of locality information.

**Table 1:** Indexing times and disk sizes

	**Dataset size**				
**Method**	**1,000**	**2,000**	**4,000**	**8,000**	**16,000**
GraphGrepSX,*l*_*p*_=4	27s	57s	1m 44s	3m 29s	7m 43s
	*2Mb*	*4Mb*	*9Mb*	*18Mb*	*37Mb*
GraphGrepSX,*l*_*p*_=10	49s	1m 44s	3m 31s	7m 09s	14m 50s
	*75 Mb*	*142 Mb*	*267 Mb*	*496 Mb*	*925 Mb*
SING,*l*_*p*_=4	34s	1m 11s	2m 17s	4m 29s	9m 12s
	*10 Mb*	*21 Mb*	*42 Mb*	*84 Mb*	*169 Mb*
SING,*l*_*p*_=10	4m 05s	8m 25s	16m 26s	32m 16s	1h 04m 39s
	*329 Mb*	*636 Mb*	*1187 Mb*	*2243 Mb*	*4279 Mb*
**BetaSearch**	**9s**	**19s**	**56s**	**2m 03s**	**4m 04s**
	***10 Mb***	***20 Mb***	***40 Mb***	***81 Mb***	***164 Mb***

The indexing times and disk sizes (italics) of each method. Indexing times were measured as the average over five repetitions. Disk sizes were measured using the POSIX “stat” command.

Subgraph-based methods (gIndex [28], FG-Index [29], and GDIndex [30]) use subgraphs as features and retain more topological information about graphs than paths due to their more complex structures. Index construction then requires time exponential to the number of nodes in each graph which also produces larger indices than those of path-based methods. These problems can be alleviated to a degree by indexing only the most frequent subgraphs [28].

Tree-based methods (TreePi [31], TreePi+*δ*[32], and CTree [33]) use subtrees as features and are purported to provide an ideal compromise between the small indices of path-based methods and the specificity of subgraph-based methods. Algorithmic operations on trees are generally more asymptotically efficient than those on graphs, in particular, subtree isomorphism can be tested in polynomial time [34]. However, previous results showed that certain path-based methods are still an order of magnitude faster in query time than existing tree-based methods [27].

GCoding [35] generates numeric representations of graphs using encodings of their adjacency matrices and cannot be classified into any of the above groups. These representations allow efficient filtering without computationally expensive graph traversals or feature enumeration. A specialised subgraph isomorphism test is used for verification [35]. While these encodings provide a compact index, expensive eigenvalue calculations are required to compute them [27].

Each of these methods can be applied to a wide variety of problems because they were designed for general graphs (i.e. graphs with an unrestricted degree and/or node count) that do not make use of the inherent structural constraints of *β*-sheets. For example, each *β*-residue has at most four neighbours: the preceding and following peptide-bonded residues and one bridge-partner located on each of the two adjacent hydrogen-bonded *β*-strands.

The problem of protein 3D substructure searching involves searching a database of protein structures for structures that contain substructures similar to a query structure and remains a significant problem in structure biology. These substructures may be relevant to biological processes such as binding sites, enzymatic function, or may be representative of a particular fold family [16,36]. Current methods for substructure searching are based on the comparison of three-dimensional coordinates between structures and use computationally complex structural alignment algorithms. Methods such as Dali [37], DaliLite [38], and SHEBA [39] align protein structures at the residue level, that is, they find one-to-one residue alignments between pairs of proteins; methods such as QPTableauSearch [40] and SATableauSearch [16] align proteins at the level of secondary structure elements (SSEs) and therefore lack residue-level specificity but are generally faster [16]. A common drawback of these methods is that exhaustive pairwise comparisons are required between the query and the each protein structure in a database. This naive approach often leads to redundant comparisons between highly similar structures or structures with no obvious match, ultimately resulting in queries requiring hours or even days to complete [16].

Recently, LabelHash [36,41] was developed primarily for the 3D substructure matching of small motifs, commonly between 4 and 15 residues. This method is unique among structural search methods in general since it uses a pre-computed index to vastly accelerate querying in a manner similar to those of graph indexing approaches. Indeed, the results in this paper show that LabelHash yields a considerable performance boost in compute time over a conventional pairwise structural alignment approach (see Results and discussion).

In this paper we describe *BetaSearch*, a method that allows fast querying of *β*-residue motifs in large datasets of protein structures. Our method leverages the natural planar constraints of *β*-sheets by indexing them as 2D matrices, known as *β*-matrices. This approach avoids the geometric, topological, and computational complexities usually involved in 3D substructure or graph querying. Furthermore, by using *β*-sheet representations independent of a 3D coordinate system, BetaSearch identifies matching *β*-residue motifs in structurally and sequentially dissimilar proteins.

## Results and discussion

We have compared the performance of BetaSearch against state-of-the-art graph indexing and 3D substructure search methods separately. The results of three case studies are also presented, which provide biologically-relevant contexts in which BetaSearch could be used.

### Comparisons with graph indexing methods

We compared BetaSearch against SING and GraphGrepSX. SING was shown to outperform existing methods in terms of query time on standard datasets of chemical compounds, protein transcription networks, protein-interaction networks, and synthetic graphs [27].

The elapsed indexing, total query, filtering, and verification times were averaged over five repetitions. SING and GraphGrepSX were run using *l*_*p*_=4 and *l*_*p*_=10, where *l*_*p*_ denotes the path length. These values were chosen by the authors of each method in their own comparisons [23,27]. We were unable to use larger *l*_*p*_ values or datasets of more than 16,000 *β*-sheets due to the memory consumption of the SING and GraphGrepSX implementations. We therefore only reported results for datasets up to and including *N* = 16,000. Accuracies of each method were not measured since each query matches at least one *β*-sheet and any non-matching *β*-sheet is excluded at the filtering and verification stages of each method.

#### Indexing

The elapsed times and disk space required for index construction are shown in Table 1.

BetaSearch recorded the fastest indexing times with a 1.9 times speed-up over the next fastest method (GraphGrepSX,*l*_*p*_=4) for the *N* = 16,000 dataset. The size of the BetaSearch indices were similar to those of SING,*l*_*p*_=4 since trimers have an effective path length of *l*_*p*_=3 and both use hash tables.

The *l*_*p*_=10 variants of SING and GraphGrepSX were slower than their *l*_*p*_=4 variants due to the increase in the number of features generated in the former case. This observation was consistent with those of general graphs [23].

GraphGrepSX,*l*_*p*_=4 generated the smallest indices by a considerable margin through the use of a suffix trees to store its indices. However, the results obtained in the following sections show that the reduction in index disk space came at a significant cost to the querying time.

Furthermore, the BetaSearch index is limited only by the size of the hard disk on which it is stored, whereas the implementations of SING and GraphGrepSX used in this study were memory-limited, requiring the entire index to be loaded into memory in order for queries to be performed.

#### Overall query times

The query time for a single query was calculated as the sum of its filtering and verification times. The time required to perform all the queries on a dataset was measured as the sum of its individual query times, shown in Table 2. These results show that BetaSearch consistently recorded the fastest querying times for all datasets by at least an order of magnitude over the next fastest method (SING,*l*_*p*_=10) and a 109 times speed-up over the baseline (GraphGrepSX,*l*_*p*_=4) for the *N* = 16,000 dataset.

**Table 2:** Overall query times (graph indexing comparisons)

	**Dataset size**				
**Method**	**1,000**	**2,000**	**4,000**	**8,000**	**16,000**
	***7205***	***14,516***	***29,101***	***58,795***	***117,415***
GraphGrepSX,*l*_*p*_=4	2m 10s	9m 13s	44m 16s	3h 19m 37s	15h 03m 54s
GraphGrepSX,*l*_*p*_=10	1m 18s	6m 12s	24m 55s	1h 50m 52s	8h 02m 31s
SING,*l*_*p*_=4	50s	3m 27s	12m 16s	52m 41s	3h 53m 45s
SING,*l*_*p*_=10	20s	1m 19s	5m 16s	23m 06s	1h 37m 49s
**BetaSearch**	**2s**	**7s**	**33s**	**2m 14s**	**8m 19s**

The overall querying times of each method were measured as the average of five repetitions. The number of queries performed on each dataset are shown in italics. A description of the query generation process is provided in the “Benchmarking datasets” section of this paper.

The trade-off between the index disk size and querying times within the SING and GraphGrepSX variants can be seen in these results where the *l*_*p*_=10 variants required four to five times as much disk space but were at least twice as fast as the *l*_*p*_=4 variants.

**Figure 2:**
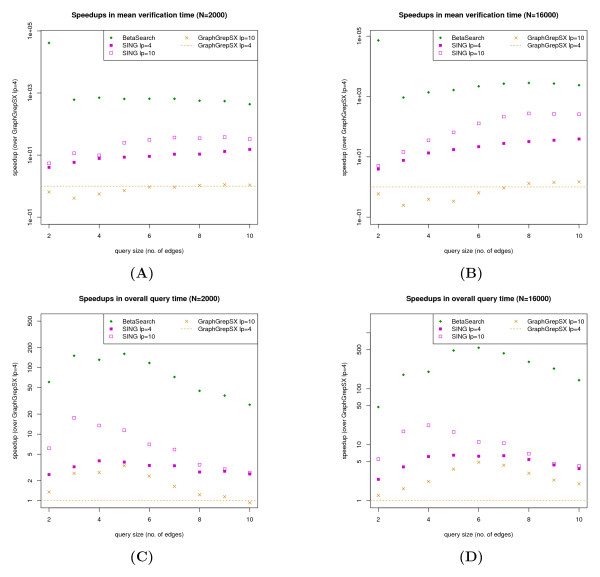
**Speedups in mean verification and overall query times.** The speedups in mean verification times and overall query times for the *n*= 2,000 and 16,000 datasets. The GraphGrepSX, *l*_*p*_=4 times were used as baselines for each plot. Mean verification times were measured as the total verification time divided by the total number of filtered candidates for each dataset.

The overall query time speedups for all query sizes were measured using the GraphGrepSX,*l*_*p*_=4 as the baseline, shown in Figure 2. Only the speedups for the *N* = 2,000 and 16,000 datasets were shown for the purposes of brevity. The speedups of each method generally tapers down after queries of approximately six to seven edges. This is an expected observation because larger *β*-sheet subgraph queries are more specific than smaller ones, resulting in fewer possible candidates and therefore a reduced filtering and verification load.

#### Filtering

The filtering time was calculated as the time required to perform filtering for all the queries of a given dataset. The precision was calculated as the total number of actual query matches divided by the total number of filtered candidates for all the queries of a given dataset. The filtering results are shown in Table 3.

**Table 3:** Filtering times and precisions (graph indexing comparisons)

	**Dataset size**				
**Method**	**1,000**	**2,000**	**4,000**	**8,000**	**16,000**
GraphGrepSX,*l*_*p*_=4	9s	40s	3m 04s	13m 30s	56m 27s
	*0.38*	*0.34*	*0.23*	*0.08*	*0.03*
GraphGrepSX,*l*_*p*_=10	10s	43s	3m 03s	13m 14s	55m 05s
	*0.92*	*0.97*	*0.97*	*0.34*	*0.20*
SING,*l*_*p*_=4	25s	1m 42s	6m 38s	28m 23s	2h 01m 40s
	*0.39*	*0.39*	*0.39*	*0.39*	*0.39*
SING,*l*_*p*_=10	13s	54s	3m 51s	16m 54s	1h 10m 32s
	*0.98*	*0.99*	*1.00*	*1.00*	*1.00*
**BetaSearch**	**2s**	**7s**	**32s**	**2m 13s**	**8m 16s**
	***1.00***	***1.00***	***1.00***	***1.00***	***1.00***

The filtering times and precisions (italics). Precision was calculated as the number of actual query matches divided by the number of filtered candidates for all queries of a given dataset.

In contrast to their indexing performances, the *l*_*p*_=10 variants of SING and GraphGrepSX generally outperformed their *l*_*p*_ = 4 variants. A larger *l*_*p*_value has more specificity and therefore results in fewer numbers of filtered candidates than a small *l*_*p*_ value, reducing the verification load. The BetaSearch and *l*_*p*_ = 10 precision values were consistently near 1.0 for all datasets and query sizes. The precision of the *l*_*p*_ = 4 variants were considerably lower than the *l*_*p*_ = 10 variants due to the aforementioned specificity limitations of smaller path lengths.

#### Verification

We measured the mean verification time as the total verification time for a dataset divided by the total number of filtered candidates for a dataset. The mean verification times were less than a second due to the relatively small query graphs involved in this study. The speedups of each method were measured using the GraphGrepSX,*l*_*p*_=4 times as the baseline and are shown in Figure 2.

BetaSearch consistently recorded the fastest verifications across all query sizes and datasets, this is because the BetaSearch verification algorithm runs in quadratic time whereas the VF2 algorithm employed by SING and GraphGrepSX was designed for general graphs and has a potential non-polynomial time complexity [21]. The largest speed-up by BetaSearch was achieved for queries with two edges, since these queries equated to individual trimers, there was no need for candidates to be verified.

### Comparisons with 3D substructure search methods

We have compared BetaSearch with LabelHash and SHEBA since they each perform residue-level matching. SHEBA was shown to be amongst the most accurate substructure search methods in recent work [16], however, LabelHash has yet to be evaluated against other methods. LabelHash and SHEBA were run using default search parameters. Comparisons with DaliLite were unable to be performed due to the majority of our queries and *β*-sheets not meeting the minimum number of residues required by DaliLite. DaliLite was shown to have accuracies comparable to SHEBA but with considerably longer compute times [16].

**Figure 3:**
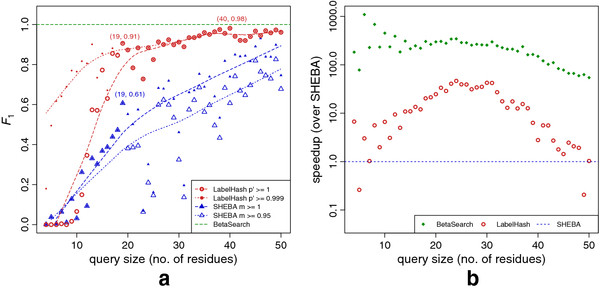
**Comparisons with 3D substructure search methods.** (**a**) The *F*_1_score versus query size, *p*^*′*^and *m* denote the hit scores at which an exact match is considered and all hits below this threshold are excluded. BetaSearch is an exact-matching method and does not produced ranked hits, it therefore yields an *F*_1_score of 1.0 for all queries. (**b**) The CPU time speedups of BetaSearch and LabelHash over SHEBA. Elapsed wallclock times were nearly identical to the CPU times and were therefore omitted.

Figure 3A shows the *F*_1_scores computed across all query sizes for each method. Exact matches for each method were considered to be those with *p*^*′*^=1 for LabelHash and *m*=1 for SHEBA. We also computed *F*_1_at *p*^*′*^≥0.999, however, the *F*_1_ at *m*≥0.999 was identical to that of *m*=1 so we instead computed *F*_1_at *m*≥0.95. BetaSearch, by virtue of inherent exact matching, produces unranked hits and consequently produces an *F*_1_ score of 1.0 for the entire query set.

LabelHash at *p*^*′*^≥0.999 clearly outperforms SHEBA on all query sizes, however, neither method performed particularly well on queries of 10 residues or less with the worst *F*_1_scores observed for queries of 4–5 residues, which have the largest number of hits amongst all query sizes (see Additional file 1: Figure S1A). Although, once the queries reach sizes of 25 residues, LabelHash maintained *F*_1_ scores of at least 0.9 since the number of possible hits closely approaches the number queries (see Additional file 1: Figure S1B).

We measured the CPU times of each method and computed the speedups over SHEBA. The wallclock times were also measured but were omitted since they were analogous to the CPU times. The CPU times of each method for the ASTRAL95 query set were measured as follows: 

• SHEBA – 239 h 25 m

• LabelHash – 33 h 17 m

• **BetaSearch – 0 h 59 m**

Figure 3B shows the speedups at each query size. BetaSearch achieved total speedups of 240 times over SHEBA and 33 times over LabelHash. The largest speedups of BetaSearch were obtained for queries of 4–15 residues, which are the sizes of commonly studied motifs [41]. The improved performance of BetaSearch and LabelHash over SHEBA can be attributed to their use of indices which removes the need to perform exhaustive pairwise comparisons for each query against the dataset. This naive approach to substructural searching can lead to query sets taking days to complete [16,40].

### Case Studies

*β*-residue motifs can contribute both to the structural and functional features of a protein. For researchers who study protein structure, BetaSearch can be a useful tool for surveying particular *β*-sheet configurations across known protein structures. The frequency of a particular motif may give an indication of its relative stability as a *β*-sheet structural element. Researchers who study functional aspects of *β*-sheets can use BetaSearch to identify similar motifs in unrelated proteins, as we demonstrate with the biotin-binding pockets of avidin and streptavidin. BetaSearch is fast with a simple, intuitive search query context that allows the researcher to efficiently make comparisons against known *β*-sheets.

A typical BetaSearch workflow involves the researcher (i) inspecting a protein structure for a *β*-sheet of interest, (ii) identifying a specific *β*-residue motif, and (iii) manually entering the amino acids in the corresponding *β*-matrix into BetaSearch. Alternatively, this workflow can be automated, allowing BetaSearch to be used in a data mining or knowledge-discovery capacity which potentially allows interesting relationships between specific amino acid configurations and protein structures or functions, which would not be intuitively revealed by manual trial-and-error querying.

To demonstrate the capabilities and potential use-cases of BetaSearch we present the results of three case studies. These were drawn from real-world examples and illustrate the role *β*-residue motifs play in the structure and function of proteins. We also provide the matches from comparative queries using BLAST to demonstrate the difference in matches between a conventional sequence-based homology search and BetaSearch (see Additional files 2, 3, and 4).

#### Case Study 1 - Synthetic motifs in the Top7 protein

**Figure 4:**
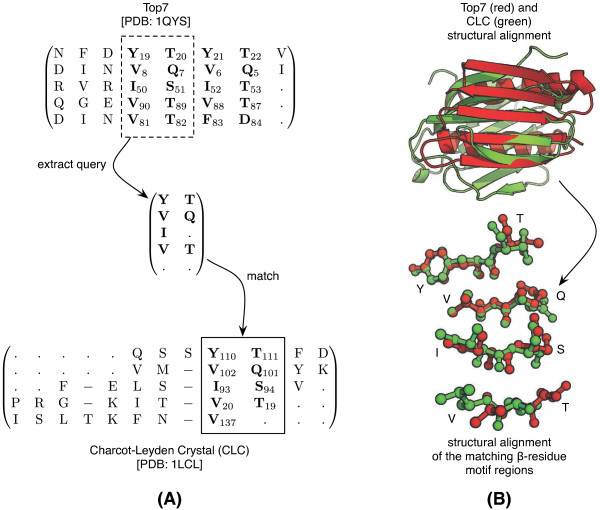
**Matching the Top7 and CLC *β*-sheets (Case Study 1).** The amphipathic query (**a**) is a characterisation of the repeating amphipathic region in the Top7 protein (bold). This query was matched only in the CLC protein [PDB: 1LCL]. A structural alignment (**b**) around the query-matching regions of both proteins where the strand and sidechain directions are conserved. The structural alignment was generated using the PyMOL “align” command: “fetch 1QYS; fetch 1LCL; align 1QYS & (resi 8+19+50+81+90), 1LCL & (resi 20+93+102+110)”.

Top7 [PDB:1QYS] is the only engineered protein (non-hypothetical) not to be derived from the sequence or structure of any other protein [42,43]. Most notably, it adopts a unique fold that has yet to be observed in nature. Its structure consists of an amphipathic *β*-sheet and two *α*-helices. Inspection of the *β*-sheet revealed a repeating *β*-residue motif (Figure 4A). Using this as a query, we wanted to discover known protein structures that possessed this putative synthetic motif. BetaSearch was used to query the PDB2011 dataset, which revealed matches only in structures of the Charcot-Leyden crystal (CLC) protein [PDB:1G86,1HDK,1LCL,1QKQ].

A structural alignment of Top7 and CLC around the matching regions of the query motif (Figure 4B) shows remarkably, that the *β*-strand topology and sidechain directions are nearly identical. This does not suggest a homology between the two proteins because Top7 is entirely synthetic. However, our findings demonstrate that the RosettaDesign [44] approach used to engineer Top7 had inadvertently reproduced a known stable *β*-residue motif *ab initio*. The CLC protein was not found in a BLAST query of Top7 chain A (see Additional file 2).

#### Case Study 2 - Biotin-binding domains

**Figure 5:**
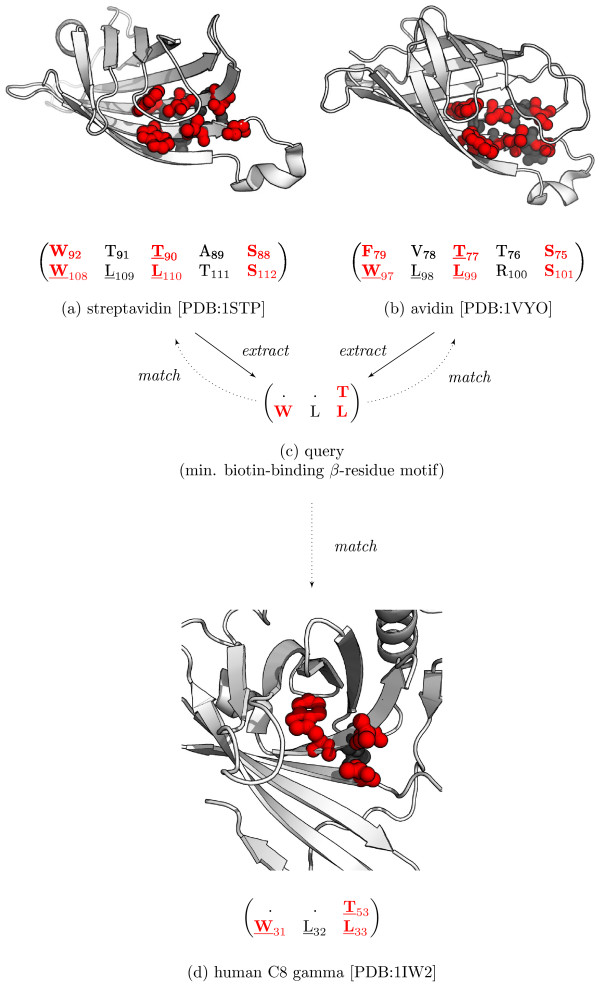
**Querying biotin-binding *β*-residue motifs (Case Study 2).** The (**a**) streptavidin and (**b**) avidin biotin-binding motifs are well conserved with the residues on the binding face coloured red. (**c**) The minimal biotin-binding *β*-residue motif used to query potential biotin-binding proteins. (**d**) The query was matched in the gamma chain of the human complement C8 protein [PDB:1IW2]. The *β*-matrices shown in (**a**), (**b**), and (**d**) have been abbreviated for clarity and are sub-matrices of larger *β*-matrices.

Streptavidin [PDB:1STP] and avidin [PDB:1VYO] are structurally and functionally similar homologous proteins that bind strongly to biotin despite having a sequence identity of less than 35%. Both proteins consist of eight antiparallel *β*-strands that fold into a *β*-barrel, inside of which forms a highly conserved biotin-binding site. The *β*-residue motifs that line this highly specific site are shown in Figures 5A and 5B. When the residues on the non-binding face of the *β*-sheet are ignored, the two motifs are differentiated by only a single residue: $W_{92}^{1\mathrm{STP}}\Leftrightarrow F_{79}^{1\mathrm{VYO}}$. The results from the corresponding BLAST query are shown in Additional file 3.

We have characterised these biotin-binding sites as a minimal, *β*-residue motif (Figure 5C). This putative motif is evolutionary conserved between the avidins and has not yet been shown to be recurrent in evolutionarily distant proteins. Using this query, BetaSearch not only identifies the structures of avidin and streptavidin, but also xenavidin—a biotin-binding protein from *Xenopus tropicalis* (frog). A number of seemingly unrelated proteins were also matched including uncharacterised proteins from *Roseovarius nubinhibens* [PDB:3BVC] and *Oceanicola granulosus* [PDB:2RG4]; and the human complement protein C8 gamma [PDB:1IW2]. The complete set of matching *β*-sheets is listed in Additional file 4.

Inspection of the uncharacterised proteins reveal a similar arrangement of residues to the known-biotin binding proteins but with less room for the ligand to bind. More tantalising is the match with the gamma subunit of human C8 which is a crucial component of the cytolytic membrane attack complex (MAC) [45]. This subunit has a characteristic lipocalin fold with a distinctive binding pocket similar to the avidins, however, the ligand target of C8 gamma remains unknown [45]. Based on the spatial similarities of this binding pocket with the biotin-binding sites of avidin, one may suggest that these proteins could have an affinity for biotin or a biotin-like compounds.

These results demonstrate that a relatively small *β*-residue motif query can be matched in unrelated proteins. This capability can be particularly useful in characterising proteins of unknown function by similarities in *β*-residue motifs to those of known function.

#### Case Study 3 - *β*-strand pairing prediction

One of the unsolved problems of tertiary structure prediction is the ability to predict the pairs of *β*-strands which are hydrogen bonded, and therefore adjacent, in a *β*-sheet [46]. Information about adjacent *β*-strands can be used to determine the overall topology of a *β*-sheet. A number of *β*-sheet topology prediction algorithms exist that are based on well-known machine learning methods [46-49].

We used BetaSearch to predict the *β*-strand pairings of the five-stranded *β*-sheet found in chain A of c-src tyrosine kinase [PDB: 1A09]. This *β*-sheet contains five strands, is non-barreled, non-bifurcated, and therefore has four native strand pairings. A score for each possible strand pair was computed as a function of the number of hits, in a BetaSearch index, obtained for each interstrand 4-mer query. The top four pairs ranked by pairing scores were considered as predictions. Strand pairing scores were computed for parallel and antiparallel orientations, as shown in Table 4. These results demonstrated that each of the native strand pairs were correctly predicted. The procedures used to perform these predictions are described in the Methods section. Our mechanisms for strand pair scoring are by no means a definitive solution to *β*-sheet topology prediction. They can, however, be used in existing algorithms such as BetaPro [47] which require preliminary *β*-strand or *β*-residue pairing scores in order for predictions to be made. A large scale evaluation of our BetaSearch-based prediction method is the topic of future work.

**Table 4:** **The predicted strand pairing scores for the 1A09 chain A *β*-sheet (Case Study 3)**

**Pair**	**Parallel score**	**Antiparallel score**	**Total score**
^∗^4,5	4.86	4.99	9.85
^∗^2,3	4.59	4.60	9.19
^∗^3,4	4.32	4.31	8.64
^∗^1,2	4.09	4.09	8.18
3,5	3.12	3.09	6.21
2,4	2.98	2.97	5.96
1,3	2.95	2.97	5.93
1,4	2.72	2.72	5.44
2,5	2.33	2.33	4.66

The strand pairing scores generated for the *β*-sheet in chain A of c-src tyrosine kinase. The top four scoring pairs (predicted) are delineated and the native pairs are denoted with an asterisk.

## Conclusion

We have described a method for indexing and querying *β*-residue motifs, called *BetaSearch*, that is at least an order of magnitude faster than state-of-the-art graph indexing methods. These speedups are achieved by indexing *β*-sheets as 2D matrices of amino acids known as *β*-matrices. This representation leverages the inherent planar structural constraints of *β*-sheets, thereby avoiding much of the computational complexity involved in querying and indexing 3D or graph representations of protein structures. BetaSearch is therefore able to achieve quadratic-time querying. Filtering precisions were close to 1.0 for all datasets and query sizes, resulting in near minimal verification time.

When compared with existing 3D substructure search methods, BetaSearch achieves a 240 times speedup over the baseline (SHEBA) and a 33 times speedup over the next fastest method (LabelHash). The demonstrated efficiency of BetaSearch lends itself well to the rapid exploration of probable motif or *β*-sheet conformations in a matter of minutes, rather than days or weeks with 3D-based methods. Furthermore, the ability of BetaSearch to perform exact matching ensures that correct hits are not missed.

Our three case studies demonstrated the utility of BetaSearch in biological contexts. We discovered that the synthetic Top7 protein shares an identical *β*-residue motif with a known naturally-occurring protein—the Charcot-Leyden crystal. A small query derived from the biotin-binding motif of avidins easily identified unrelated biotin-binding proteins and is suggestive of biotin-binding in others including the gamma subunit of the human C8 complement protein. BetaSearch, with its ability to identify functional similarity from unrelated proteins can potentially help characterise the proteins in the PDB with unknown function. We also demonstrated how BetaSearch could be used to predict strand pairing in *β*-sheets, which could help reduce the search space of more complex supersecondary or tertiary structure prediction tasks. Although our work has focused on substructural motifs in *β*-sheets, our algorithm can be modified to perform querying of any substructural motif involving pairwise interactions, such as the well-characterised hydrogen-bond pairings in helices and turns. Indeed, this is an avenue of development we are currently exploring.

It is our intention for BetaSearch to be used by protein researchers to supplement conventional sequence and structural search methods. For example, the efficiency of the BetaSearch filtering and verification algorithms introduces the possibility for their use as a rapid “first-pass” filter to improve the querying performance of other methods. Such an application would be non-trivial to develop but could potentially reduce conventional structural query times from hours to minutes.

## Findings

The pseudocode for each of the algorithms described in this section is provided in the Supplementary Materials.

### Trimers

A *trimer* is a path of three amino acids in a *β*-matrix configured in the shape of an ‘L’ (an L-trimer), vertically in the same column (a V-trimer), or horizontally in the same row (an H-trimer). Trimers are the features by which *β*-matrices are indexed in BetaSearch. An example of the trimer extraction process is shown in Additional file 1: Figure S2.

A trimer *t* has a number of attributes that encode its configuration and location within a *β*-matrix: 

• *t*.seq: a three letter string of residues spanned by the trimer where

(4) $$\begin{array}{l} \;\text{if}\;\text{t.}\text{seq}=\text{“abc”}\\ \;\text{then}\;\text{t.}\text{seq}[0]\to \text{“a”},\;\text{t.}\text{seq}[1]\to \text{“b”},\;\text{t.}\text{seq}[2]\to \text{“c”}. \end{array}$$

• *t*.class: an integer representing the *class* of the trimer, defined as

(5) $$\begin{array}{l} \mathrm{t.}\text{class}=\left\{\begin{array}{l} 1\;\;\text{if}\;t\;\text{is an L-trimer}\\ 3\;\;\text{if}\;t\;\text{is a V-trimer and}\;\mathrm{t.}\text{seq}[0]\neq \mathrm{t.}\text{seq}[2]\\ 5\;\;\text{if}\;t\;\text{is an H-trimer and}\;\mathrm{t.}\text{seq}[0]\neq \mathrm{t.}\text{seq}[2]\\ 15\;\;\;\text{if}\;t\;\text{is a V-trimer and}\;\mathrm{t.}\text{seq}[0]=\mathrm{t.}\text{seq}[2]\\ 31\;\;\;\text{if}\;t\;\text{is an H-trimer and}\;\mathrm{t.}\text{seq}[0]=\mathrm{t.}\text{seq}[2]. \end{array}\right) \end{array}$$

• *t*.id: a (*t*.class,*t*.seq) tuple.

• *t*.orient: an integer value such that *t*.orient$\in \left\{0,1,2,3\right)\}$. These values describe the possible orientations of a trimer and were chosen to allow the calculation of x- and y-axis trimer reflections using the bitwise-XOR (‘⊕’) operator. Orientation reflections are calculated as

(6) $$\begin{array}{c} t^{\prime }.\text{orient}=\left\{\begin{array}{cc} \mathrm{t.}\text{orient}⊕1 & \;\text{if reflected in the y-axis}\\ \mathrm{t.}\text{orient}⊕2 & \;\text{if reflected in the x-axis} \end{array}\right) \end{array}$$

where *t’* is the reflection of *t*. Additional file 1: Figure S2 shows how trimer orientations are determined for each trimer class.

*t*.eq-orients: an integer that encodes the equivalent orientations of *t*.orient, defined as

(7) $$\begin{array}{l} \text{t.}\text{eq-orients}=\left\{\begin{array}{l} 2^{\text{t.}\text{orient}}\;\;\text{if}\;\text{t.}\text{class}=1\\ 3\;\;\;\;\;\text{if}\;\text{t.}\text{class}=3\;\text{and}\;\text{t.}\text{seq}[0]<\text{t.}\text{seq}[2]\\ 12\;\;\;\;\text{if}\;\text{t.}\text{class}=3\;\text{and}\;\text{t.}\text{seq}[0]>\text{t.}\text{seq}[2]\\ 5\;\;\;\;\;\text{if}\;\text{t.}\text{class}=5\;\text{and}\;\text{t.}\text{seq}[0]<\mathrm{t.}\text{seq}[2]\\ 10\;\;\;\;\text{if}\;\text{t.}\text{class}=5\;\text{and}\;\text{t.}\text{seq}[0]>\text{t.}\text{seq}[2]\\ 15\;\;\;\;\text{otherwise,} \end{array}\right) \end{array}$$

such that

(8) $$\begin{array}{l} \text{t.}\text{eq-orients}\;\text{\&}\;i=\left\{\begin{array}{l} 1\;\text{if orientation}\;i\;\text{is equivalent to}\;\text{t.}\text{orient}\\ 0\;\text{otherwise,} \end{array}\right) \end{array}$$

where ‘<’ and ‘>’ are the lexicographic less-than and greater-than operators; and ‘&’ is the bitwise-AND operator. The equivalent orientations of a trimer are encoded using bitmasks such that orientation *i* is equivalent to *t*.ORIENT if the *i*^th^bit of *t*.class is set to 1. The *t*.class value for each type of trimer is an encoding of the minimum *t*.class value for the class.

• *t*.row: the row coordinate of *t*.seq[1] within its *β*-matrix.

• *t*.col: the column coordinate of *t*.seq[1] within its *β*-matrix.

•*t*.coord: a (*t*.row,*t*.col) tuple.

•*t*.span1, *t*.span2: the row (*t*.row-span) or column spans (*t*.col-span) of a trimer, depending on the trimer class. Each span is an ordered tuple (*i*,*j*) where *i* is the coordinate of *t*.seq[1] and *j* is the coordinate of either *t*.seq[0] or *t*.seq[2], depending on the trimer type. L-trimers have one row span and one column span, V-trimers have two row spans, and H-trimers have two column spans. Examples of the spans for each trimer are shown in Additional file 1: Figure S3.

### Index construction

BetaSearch uses three indices: D, R, and C.

• D is an inverted index that maps each trimer *id* to the set of *β*-matrices in which they are contained, defined as

(9) D[id]↦{b∈B:id∈b.trimer-ids}

where *B* is the set of *β*-matrices in the dataset.

• R maps a compound key $\kappa_{R}$ to a trimer *t*, defined by

(10) $$\begin{array}{l} \;R[\kappa_{R}]\mapsto t\\ \;\text{where}\;\;\kappa_{R}=\left(\mathrm{t.}\text{matrix-id},\;\mathrm{t.}\text{id},\;\mathrm{t.}\text{eq-orients},\;\right)\\ \;\left(\mathrm{t.}\text{coord},\;\mathrm{t.}\text{row-span}\right) \end{array}$$

such that *class*∉{3,15}.

• C maps a compound key $\kappa_{C}$ to a trimer *t*, defined by

(11) $$\begin{array}{l} \;C[\kappa_{C}]\mapsto t\\ \;\text{where}\;\;\kappa_{C}=\left(\mathrm{t.}\text{matrix-id},\;\mathrm{t.}\text{id},\;\mathrm{t.}\text{eq-orients},\;\right)\\ \;\left(\text{t.}\text{coord},\;\text{t.}\text{col-span}\right) \end{array}$$

such that *t*.class∉{5,31}.

L-trimers are indexed in R and C; whereas H-trimers are indexed only in C because they do not contain any row spans, conversely, V-trimers are indexed only in R because they do not contain any column spans. The Build-Indices procedure in Additional file 1: Algorithm S1 describes the index construction algorithm.

#### Time complexity

Each entry in a *β*-matrix is the intersection of at most six trimers: four L-trimers (one in each of the four orientations), one V-trimer, and one H-trimer. Build-Indices runs in *O*(6*mn*) time where *m* is the maximum number of residues in a *β*-matrix and *n* is the number of *β*-matrices.

### Filtering

A query is a well-formed *β*-matrix *Q*. Preprocessing *Q* requires the following steps: 

1. Enumerate the query trimers and storing them in *Q*.trimers.

2. Store the corresponding trimer IDs in *Q*.trimer-ids.

Partially matching candidates are obtained by pruning the *β*-matrices in D using two filters. The First-Filter procedure, described in Additional file 1: Algorithm S2, prunes the *β*-matrices in D that do not contain the entire set of trimer IDs in *Q*.trimer-ids.

*β*-matrices are indexed in a single arbitrary orientation, therefore a procedure for comparing a query against all orientations of a candidate is required. The naive approach enumerates and stores the x- and y-axes reflections of each *β*-matrix, effectively tripling the index size. Alternatively, the x- and y-axes reflections of the query are enumerated and compared with a candidate, effectively tripling the filtering time.

We have developed an algorithm that prunes invalid candidates without enumerating reflections of the candidate or the query. The algorithm is implemented as the Second-Filter procedure described in Additional file 1: Algorithm S3, where only the candidates congruent to *Q* are retained from *C*_1_. A query *Q* is congruent to a candidate *c* if

(12) ⋂q∈Q.trimerst∈c.trimers{q.orient⊕t.orient:t.id=q.id}≠∅where`⊕′is the bitwise-XOR operator.

The Congruent procedure defined in Additional file 1: Algorithm S3 implements Equation 5 using bitwise operations that enable constant-time set unions and intersections.

#### Time complexity

The First-Filter algorithm runs in $O\left(|p_{\min}|\cdot \right)$|*Q.*trimer-ids|) time where $\left|p_{\min}\right|$ is the cardinality of the smallest postings set and |*Q.*trimer-ids| is the number of unique trimer ids in the query. A postings set is a set in the index of *β*-matrices containing a particular query trimer. The Second-Filter algorithm runs in $O\left(\left|\mathrm{Q.}\text{trimer-ids}|\cdot |C_{1}\right|\right)$ time where |*C*_1_| is the number of candidates returned by First-Filter.

### Verification

Most graph indexing methods use the VF2 [20] or Ullmann [19] algorithms for candidate verification. Other methods use algorithms optimised for the data structures of their features and indices.

Constraining *β*-graphs to the simpler structures of *β*-matrices has enabled us to develop a quadratic time candidate verification algorithm that does not rely on subgraph isomorphism tests.

A graph *G* of the query is constructed, in which each vertex is a trimer *q*∈*Q.*trimers and each edge *e*= $\left(q_{\mathrm{src}},q_{\mathrm{des}}\right)$ indicates a span overlap (i.e. *t*.col-span or *t*.row-span) between adjacent query trimers *q*_src_ and *q*_des_. The algorithm to construct a query graph is implemented in the Make-Query-Graph procedure described in Additional file 1: Algorithm S4.

The remainder of the verification algorithm attempts to find a subgraph of a candidate *c* that matches *G* by matching each pair $\left(q_{\mathrm{src}},q_{\mathrm{des}}\right)$ to a pair $\left(t_{\mathrm{src}},t_{\mathrm{des}}\right)\in \mathrm{c.}\text{trimers}$, where a match between pairs is defined by

(13) $$\begin{array}{l} \text{Match-Pairs}\left(q_{\mathrm{src}},q_{\mathrm{des}},t_{\mathrm{src}},t_{\mathrm{des}}\right)=\\ \;\text{Rel-Orient}\left(q_{\mathrm{src}},q_{\mathrm{des}}\right)\Leftrightarrow \text{Rel-Orient}\left(t_{\mathrm{src}},t_{\mathrm{des}}\right)\\ \;\wedge \;\text{Overlaps}\left(q_{\mathrm{src}},q_{\mathrm{des}}\right)\Leftrightarrow \text{Overlaps}\left(t_{\mathrm{src}},t_{\mathrm{des}}\right)\\ \;\wedge \;\text{Overlap-Type}\left(q_{\mathrm{src}},q_{\mathrm{des}}\right)\\ \;\Leftrightarrow \text{Overlap-Type}\left(t_{\mathrm{src}},t_{\mathrm{des}}\right)\\ \;\wedge \;\text{Overlap-Span-Nums}\left(q_{\mathrm{src}},q_{\mathrm{des}}\right)\\ \;\Leftrightarrow \text{Overlap-Span-Nums}\left(t_{\mathrm{src}},t_{\mathrm{des}}\right) \end{array}$$

and a match between a query *Q* and a candidate *c* occurs if

(14) $$\begin{array}{c} \underset{q_{\mathrm{src}},\;q_{\mathrm{des}}\;\in \;G}{\wedge }\;\left(\underset{t_{\mathrm{src}},\;t_{\mathrm{des}}\;\in \;c}{\vee }\text{Match-Pairs}(q_{\mathrm{src}},q_{\mathrm{des}},t_{\mathrm{src}},t_{\mathrm{des}})\;\right) \end{array}$$

The keys by which trimers are indexed in R and C contain their locations and geometric configurations within a candidate, allowing Match-Pairs to be tested in constant-time. Match-Pairs is implemented using the procedures defined in Additional file 1: Algorithm S8. The Verify-Candidate procedure in Additional file 1: Algorithm S6 describes our algorithm for verifying a single candidate.

#### Time complexity

The Make-Query-Graph procedure runs in $O(\left|\mathrm{Q.}\text{trimers}\right|)$ time where |*Q.*trimers| is the number of trimers in the query. The Verify-Candidate procedure runs in $O({\left|\mathrm{Q.}\text{trimers}\right|}^{2})$ time, which is called by the Verify procedure in Additional file 1: Algorithm S7 to verify each filtered candidate in *C*_2_. Therefore, the overall time complexity of our candidate verification algorithm is $O\left(\left|\mathrm{Q.}\text{trimers}\right|+\left|C_{2}\right|\cdot {\left|\mathrm{Q.}\text{trimers}\right|}^{2}\right)$.

### *β*-strand pairing prediction

For Case Study 3, we constructed a BetaSearch index from the ASTRAL95 dataset, as per the 3D substructure search comparisons. The secondary structures for the *β*-sheet were using DSSP. Each *β*-strand sequence was delineated as contiguous substrings of “E” secondary structure assignments. Scores for each possible *β*-strand pairing (*i*,*j*) were computed as follows: 

1. Let I be the index generated from the ASTRAL95 dataset.

2. Let *S*^*P*^and *S*^*A*^be the strand pairing score matrices for the parallel and antiparallel strand pairs, respectively.

3. Let *M* be a matrix where *M*_*ij*_contains the number of 4-mers between strands *i* and *j*. We define a 4-mer as a single occurrence of two-consecutive bridge pairings. For example, the *β*-matrix $\left(\begin{array}{ccc} A & B & C\\ D & E & F \end{array}\right)$ has the 4-mers – $\left(\begin{array}{cc} A & B\\ D & E \end{array}\right)$ and $\left(\begin{array}{cc} B & C\\ E & F \end{array}\right)$.

4. For each strand pair (*i*,*j*): 

(a) For each alignment *a* of *j* on *i*: 

(i) For each 4-mer *m* of *a*: 

(A) Let *h*_*P*_and *h*_*A*_be the number of hits returned from querying I for the parallel and antiparallel orientations of *m*, respectively.Set $S_{\text{ij}}^{P}\leftarrow S_{\text{ij}}^{P}+h_{P}$Set $S_{\text{ij}}^{A}\leftarrow S_{\text{ij}}^{A}+h_{A}$Set $M_{\text{ij}}\leftarrow M_{\text{ij}}+1$

(b) Let $\mathrm{seq}\text{\_}\mathrm{sep}\left(i,j\right)$ be the number of residues separating strands *i* and *j*.Set $S_{\text{ij}}^{P}\leftarrow \frac{S_{\text{ij}}^{P}}{M_{\text{ij}}}-\log\left[\mathrm{seq}\text{\_}\mathrm{sep}\left(i,j\right)\right]$Set $S_{\text{ij}}^{A}\leftarrow \frac{S_{\text{ij}}^{A}}{M_{\text{ij}}}-\log\left[\text{seq\_sep}\left(i,j\right)\right]$

The total score for a strand pair was defined as

(15) $$\text{score}\left(i,j\right)=S_{\text{ij}}^{P}+S_{\text{ij}}^{A}$$

### Evaluation

The graph indexing methods were evaluated according to their filtering, verification, and overall query times. The sizes of the indices generated by each method were measured using the POSIX “stat” command. The filtering precision was calculated as the total number of hits divided by the total number of filtered candidates for all queries of a given dataset size.

A “hit” for a query against a *β*-sheet occurs when it contains (or is) an exact match of the query structure. Unlike BetaSearch, LabelHash and SHEBA perform approximate matching and return a list of hits ranked by a structural similarity score. For LabelHash, this is a statistically-determined *p*-value where a low value indicates a close match. SHEBA ranks hits according to the *m*-value which is the number of aligned residues between a query and a result, divided by the number of residues in the larger of the two structures. For simplicity, we denote the LabelHash score for a hit as *p*^*′*^=1−*p*. These scores need to be thresholded in order to obtain exact matches so that all hits with scores below a given threshold are ignored and those above the threshold are assigned the same rank. Once a suitable hit score threshold is chosen, the querying accuracy of each method can be computed by counting a hit as a true positive (*TP*) if the query is exactly matched within a *β*-sheet, a false positive (*FP*) if it is not, or a false negative (*FN*) if a correct *β*-sheet hit is absent from the list of results. We can then calculate the *recall*, as

(16) $$\text{recall}=\frac{\text{TP}}{\text{TP}}+\text{FN}$$

which denotes the proportion of exactly-matching *β*-sheets out of all correct *β*-sheet hits. and the *precision*, as

(17) $$\text{precision}=\frac{\text{TP}}{\text{TP}}+\text{FP}$$

which denotes the proportion of correct *β*-sheet hits in a list of hits. These two measures are commonly used to evaluate conventional document retrieval systems [24], as well as protein structural search methods [16]. The *F-score*, defined as

(18) $$F_{\beta }=\left(1+\beta^{2}\right)\cdot \frac{\text{precision}\cdot \text{recall}}{\beta^{2}\cdot \text{precision}+\text{recall}}$$

is the harmonic mean of the precision and recall values. It provides a convenient way of evaluating the query precision and recall as a single value. The *β*parameter allows emphasis to be placed on precision or recall depending on the query performance goals. We used the *F*_1_ score in our 3D substructure search comparisons as a measure of query accuracy.

### Datasets

For the graph indexing comparisons, the January 3, 2011 (PDB2011) snapshot of the PDB was used to generate a dataset of 209,127 *β*-sheets. A number of PDB files were excluded due to discrepancies in their content [50]. *β*-sheets exhibiting poor planarity, such as those with significantly pronounced twisting or curvature, were also excluded.

The DSSP [51] algorithm was used to assign secondary structures to each PDB file. Residues with a secondary structural assignment of “E” or “b” were considered to form part of a *β*-strand. DSSP also assigns bridge partner relationships between residues on adjacent *β*-strands, which were used to determine the bridge edges in *β*-graphs.

We used Pro-Origami [52] to generate *β*-graphs and a topological sort was used to generate the *β*-matrix from the peptide and bridge edges of each *β*-graph.

Subsets containing 1,000; 2,000; 4,000; 8,000; and 16,000 *β*-sheets were randomly selected from the dataset. These sizes were used in previous benchmarks [23,27]. GraphGrepSX and SING were unable to be run on datasets of 32,000 or more due to the memory consumption of their respective implementations, we therefore restricted the sizes of our datasets accordingly.

Each *β*-graph was preprocessed by inserting “dummy” nodes in place of a labelled edge. Each dummy node was labelled with either a “b” to denote a bridge edge or a “z” to denote a peptide edge in order to avoid conflict with the labels of residue nodes. Preprocessing was required because edge-labelled graphs were not supported by SING or GraphGrepSX.

Queries were generated in the same manner as in previous benchmarks [23]. A query was created from each *β*-graph by randomly selecting a root node and performing a random breadth-first traversal until the query obtained the degrees *d* such that 2≤*d*≤10. Queries were not generated from *β*-graphs with insufficient edges. Each query contained a single wildcard node that matched any amino acid. To enable wildcard matching on all methods, each query was repeated by replacing the wildcard node with each of the 20 amino acids. The *β*-matrices for each query were generated for use by BetaSearch. The total numbers of queries generated for each query size are shown in Additional file 1: Table S1.

For the case studies, we generated the required indices from all the *β*-matrices in the PDB2011 dataset. Each *β*-matrix was assigned a sheet identifier of the format “Â¡PDB ID¿Â¡chain ID¿_SHEET_Â¡number¿”. For example, the sheet ID of the first *β*-matrix in chain A of ubiquitin [PDB:1UBQ] is “1UBQA_SHEET_000”.

For the 3D substructure search comparisons, the ASTRAL SCOP 1.75A 95% sequence identity non-redundant dataset [53] of protein structures (ASTRAL95) were used. *β*-sheets were extracted and filtered as per the graph indexing datasets and a total of 29,341 *β*-sheets were obtained. A subset of 26,669 *β*-sheets containing between 4 and 50 residues, inclusive, were used as queries. The *β*-matrices corresponding to each *β*-sheet were generated and used with BetaSearch.

### Implementation

The graph indexing comparisons were performed on a 2.66 Ghz Intel Nehalem 8-core processor with 48 GB of main memory running CentOS. The source code to SING and GraphGrephSX were provided by the authors of their respective publications. All methods were implemented in C++, compiled using g++ version 4.3, and depend on the Boost C++ version 1.42.0 libraries [54]. BetaSearch additionally requires Redis [55] version 2.0.4 and the official Redis C headers [56].

The indices in SING and GraphGrepSX were implemented as modified C++ STL “std::map” containers in the memory spaces of their respective processes. In contrast, BetaSearch stores its indices using Redis hash tables that are stored in (disk-based) virtual memory and operates external to BetaSearch as a concurrent process. It is therefore subject to interprocess communication overhead during filtering and indexing, which are included in our experimental timings.

The 3D substructure search comparisons were performed on the same platform with 8 GB of main memory. BetaSearch was re-implemented in Python 2.7 using the Whoosh Python Search Library [57]. LabelHash 1.0.2 [58] and SHEBA 3.1.1 [59] were downloaded and used. The LabelHash index was built from the PDB coordinates of all the ASTRAL95 *β*-sheets, which were extracted from their original structures using ProDy [60]. The BetaSearch index was built from the *β*-matrix representations of each *β*-sheet.

### Structural renderings and alignments

We used PyMOL [61] to generate 3D renderings and structural alignments of proteins.

## Availability and requirements

• **Project name:** BetaSearch

•**Project homepage:**http://www.csse.unimelb.edu.au/∼hohkhkh1/betasearch

•**Operating system(s):** Ubuntu Linux 11.10+ ( http://www.ubuntu.com)

•**Programming language(s):** Python

•**Other requirements:** A complete listing of Python module dependencies is provided on the project homepage.

•**License:** None

•**Any restrictions to use by non-academics:** BetaSearch can be used free-of-charge by non-academics, provided appropriate citation and credit is given to the authors of this publication.

## Competing interests

The authors declare that they have no competing interests.

## Authors’ contributions

HH, GG, and KR contributed to the design of the algorithms. HH implemented the algorithms and evaluation software, performed the experimental analyses, and prepared the manuscript and figures. MJK contributed to the evaluation of the case studies. All authors read and approved the final manuscript.

## Funding

This work was supported by a Victorian Life Sciences Computation Initiative (VLSCI) [grant number VR0127] on its Peak Computing Facility at the University of Melbourne, an initiative by the Victorian Government. HH is supported by a NICTA PhD scholarship. NICTA (National ICT Australia) is funded by the Australian Government’s Department of Communications; Information Technology and the Arts; Australian Research Council through Backing Australia’s Ability; ICT Centre of Excellence programs.

## Supplementary Material

Additional file 1 **Supplementary materials (supplement.pdf).** This PDF file contains additional information about our experiments as well as the pseudocode for each algorithm referenced in this paper. Click here for file

Additional file 2 **Results from a BLAST query of Top7 chain A (1QYSA-BLAST-results.txt).** This plain text file contains the accession numbers of the protein sequences obtained from a BLAST query of Top7 [PDB:1QYS] chain A. The query parameters are defined in this file. Click here for file

Additional file 3 **Results from a BLAST query of streptavidin chain A (1STPA-BLAST-results.txt).** This plain text file contains the accession numbers of the protein sequences obtained from a BLAST query of streptavidin [PDB:1STP] chain A. The query parameters are defined in this file. Click here for file

Additional file 4 **Matching*β*-sheets from Case Study 2 (case-study-2-matches.txt).** This plain text file contains all the matching *β*-sheets in our dataset from the biotin-binding *β*-residue motif query defined in Figure 5C. Click here for file
