# Supplementary material for: BetaSearch: a new method for querying β-residue motifs
Source: BMC Res Notes. 2012 Jul 30;5:391. doi: 10.1186/1756-0500-5-391 (PMC3532365; doi:10.1186/1756-0500-5-391)
Supplement: Additional file 1 — Supplementary materials (supplement.pdf). This PDF file contains additional information about our experiments as well as the pseudocode for each algorithm referenced in this paper. [file 1756-0500-5-391-S1.pdf]

BetaSearch: a new method for querying β-residue motifs
Supplementary Materials

Hui Kian Ho* Graeme Gange Michael J. Kuiper Kotagiri Ramamohanarao

April 30, 2012

This document contains descriptions of the algorithms and query counts that were omitted from our manuscript entitled "*BetaSearch: a new method for querying β-residue motifs*", for the purposes of brevity.

Should you have any questions regarding the manuscript or items in this document, you may contact me via email: `hohkhkh1@csse.unimelb.edu.au`.

1 3D substructure search queries

Figure 1 shows the histograms of the number queries and hits for each query size (every two query sizes are binned), generated from the ASTRAL95 dataset.

2 Exploratory querying of Top7

We translated each amino acid of our entire dataset of 209,127 β-matrices (from the PDB2011 dataset) to a reduced alphabet according to their hydrophobic ("h") or hydrophilic ("p") properties. We used a conventional amino acid grouping scheme [1] (SM-Table 2) to determine the amino acid translations. We subsequently formulated an "amphipathic" query as

$$\begin{pmatrix} \mathrm{h} & \mathrm{p} & \mathrm{h} & \mathrm{p} \\ \mathrm{h} & \mathrm{p} & \mathrm{h} & \mathrm{p} \\ \mathrm{h} & \mathrm{p} & \mathrm{h} & \mathrm{p} \\ \mathrm{h} & \mathrm{p} & \mathrm{h} & \mathrm{p} \end{pmatrix} \tag{1}$$

This query returned 259 matching β-matrices from 116 protein structures, in which the Top7 protein [PDB:1QYS] was found with a sheet ID of 1QYSA_SHEET_000.

The Top7 protein is of particular interest to protein researchers because it was the first (and currently, the only) engineered protein not to be derived from the structure or sequence of any other known protein [2]. The protein was designed using the RosettaDesign suite [3] *ab initio* then experimentally expressed and crystallised. The x-ray structure exhibited a remarkable 1.2Å similarity to the *in silico* model.

*corresponding author, `hohkhkh1@csse.unimelb.edu.au`

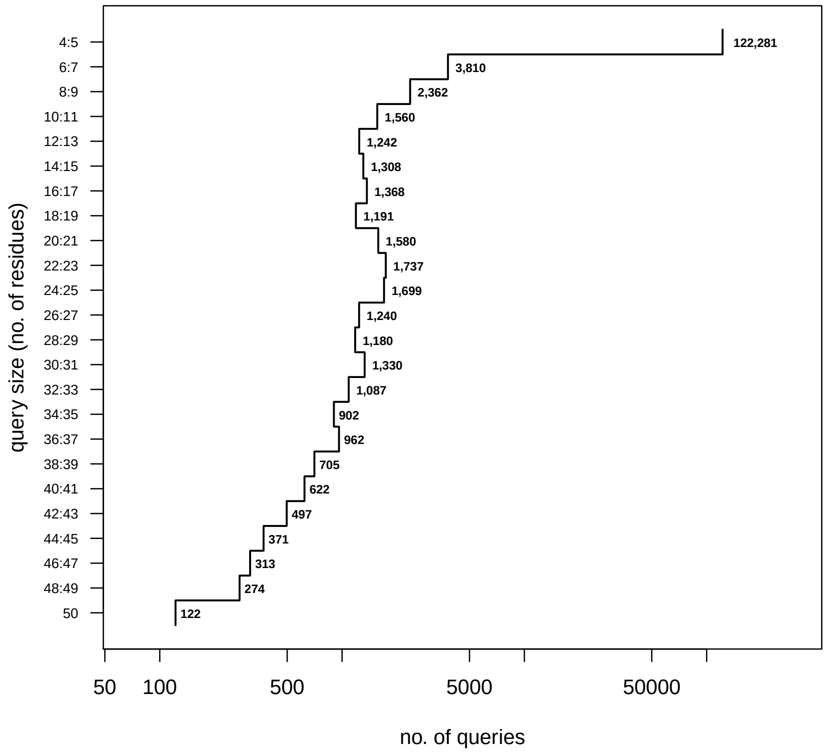

(a)

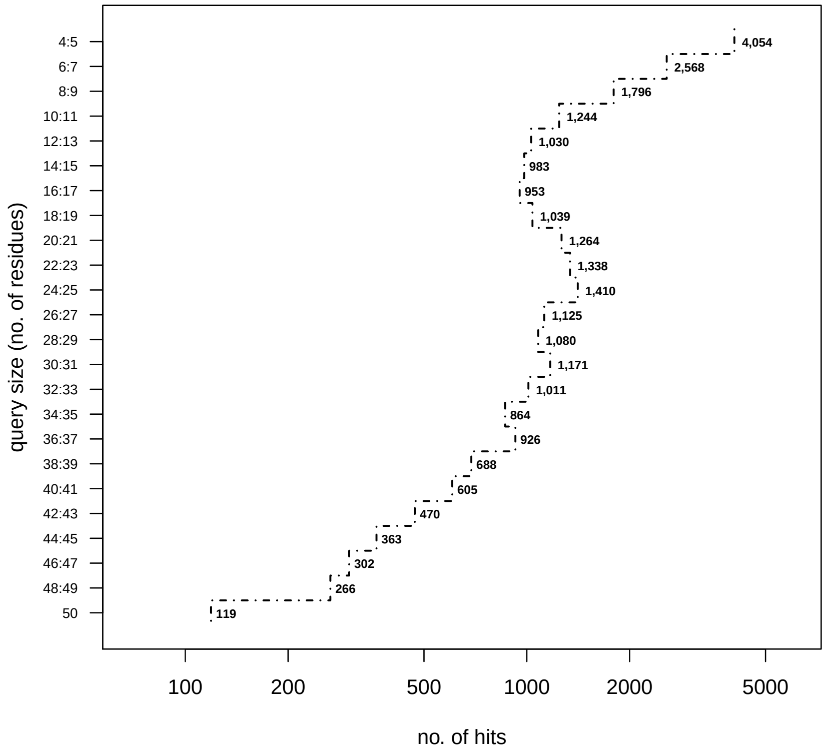

(b)

Figure 1: Histograms of the number of queries and hits for each query size in the ASTRAL95 dataset.

L-trimers

	x_0	x_1	x_2
y_0	.	.	.
y_1	.	b	c
y_2	.	a	.

	L-trimer 1	L-trimer 2	L-trimer 3	L-trimer 4
	. . .	. . .	. a .	. a .
	. b c	c b .	. b c	c b .
	. a .	. a .	. . .	. . .
$t.\text{SPAN}1$ (row-span):	(x_1, x_2)	(x_1, x_2)	(x_1, x_0)	(x_1, x_0)
$t.\text{SPAN}2$ (col-span):	(x_1, x_2)	(x_1, x_0)	(x_1, x_2)	(x_1, x_0)

V-trimers

			symmetric
	. a .	. c .	. a .
	. b .	. b .	. b .
	. c .	. a .	. a .
$t.\text{SPAN}1$ (row-span):	(x_1, x_0)	(x_1, x_2)	$(x_1, x_0 \vert x_2)$
$t.\text{SPAN}2$ (row-span):	(x_1, x_2)	(x_1, x_0)	$(x_1, x_0 \vert x_2)$

H-trimers

			symmetric
	. . .	. . .	. . .
	a b c	c b a	a b a
	. . .	. . .	. . .
$t.\text{SPAN}1$ (col-span):	(x_1, x_0)	(x_1, x_2)	$(x_1, x_0 \vert x_2)$
$t.\text{SPAN}2$ (col-span):	(x_1, x_2)	(x_1, x_0)	$(x_1, x_0 \vert x_2)$

Figure 2: Trimer span assignments. The column and row indices are shown for the left-most L-trimer. The spans of symmetrictrimers are interchangeable such that $t.\text{SPAN}1 = t.\text{SPAN}2$.

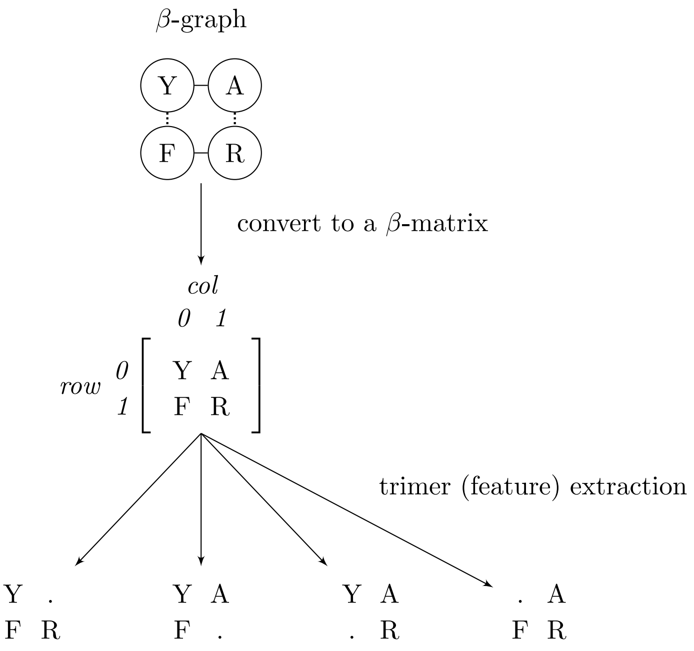

t.ID:	(1,YFR)	(1,FYA)	(1,RAY)	(1,ARF)
t.CLASS:	2	0	1	3
t.CLASS:	4	1	2	8
t.ROW:	1	0	0	1
t.COL:	0	0	1	1
t.SPAN1:	(1,0)	(0,1)	(0,1)	(1,0)
t.SPAN2:	(0,1)	(0,1)	(1,0)	(1,0)

Figure 3: An example of the trimer extraction process for a single β-graph.

Algorithm 1: BUILD-INDICES constructs the $\mathcal{D}$, $\mathcal{C}$, and $\mathcal{R}$ indices from a set of β-matrices B.

BUILD-INDICES(B)

1. For each β-matrix b in the dataset B.
   (a) For each residue r in b and for each t in T where T is the set of the trimers originating from r.
      i. If t is not a V-trimer, set $\mathcal{R}[\kappa_{\mathcal{R}}] \leftarrow \mathcal{R}[\kappa_{\mathcal{R}}] \cup t$
      ii. If t is not an H-trimer, set $\mathcal{C}[\kappa_{\mathcal{C}}] \leftarrow \mathcal{C}[\kappa_{\mathcal{C}}] \cup t$
      iii. Set $\mathcal{D}[t.\textsc{id}] \leftarrow \mathcal{D}[t.\textsc{id}] \cup t.\textsc{matrix-id}$
2. Return $\mathcal{D}$, $\mathcal{R}$, and $\mathcal{C}$.

Algorithm 2: FIRST-FILTER returns the set of β-matrices that contain all the trimer IDs in the query.

FIRST-FILTER($Q, \mathcal{D}$)

1. Set $C_1 \leftarrow \bigcap_{id \,\in\, Q.\textsc{trimer-ids}} \mathcal{D}[id]$

   where $\mathcal{D}[id] \rightarrow \{c \in D : id \in c.\textsc{trimer-ids}\}$.
2. Return C_1

Algorithm 3: SECOND-FILTER returns the set of β-matrices that are congruent to the query. The '+', '$\ll$', '$\oplus$', and '&' operators in CONGRUENT are the bitwise operators: OR, left-shift, XOR, and AND.

CONGRUENT(Q, c)
begin
  $\psi_c \leftarrow 15$;   // Initialise with the symmetric V-trimer class number.
  foreach $id \in Q.\textsc{trimer-ids}$ **do**
    $\psi_q \leftarrow 0$
    foreach $q \in \{t \in Q.\textsc{trimers} : t.\textsc{id} = id\}$ **do**
      $\psi_c \leftarrow \psi_q + (q.\textsc{class} \mod 16) \ll (q.\textsc{orient} \oplus t.\textsc{orient})$
    $\psi_c \leftarrow \psi_c \;\&\; \psi_q$
  if $\psi_c = 0$ **then**
    return False
  else
    return True

SECOND-FILTER(Q, C_1)
begin
  $C_2 \leftarrow \{c \in C_1 : \textsc{Congruent}(Q, c)\}$
  return C_2

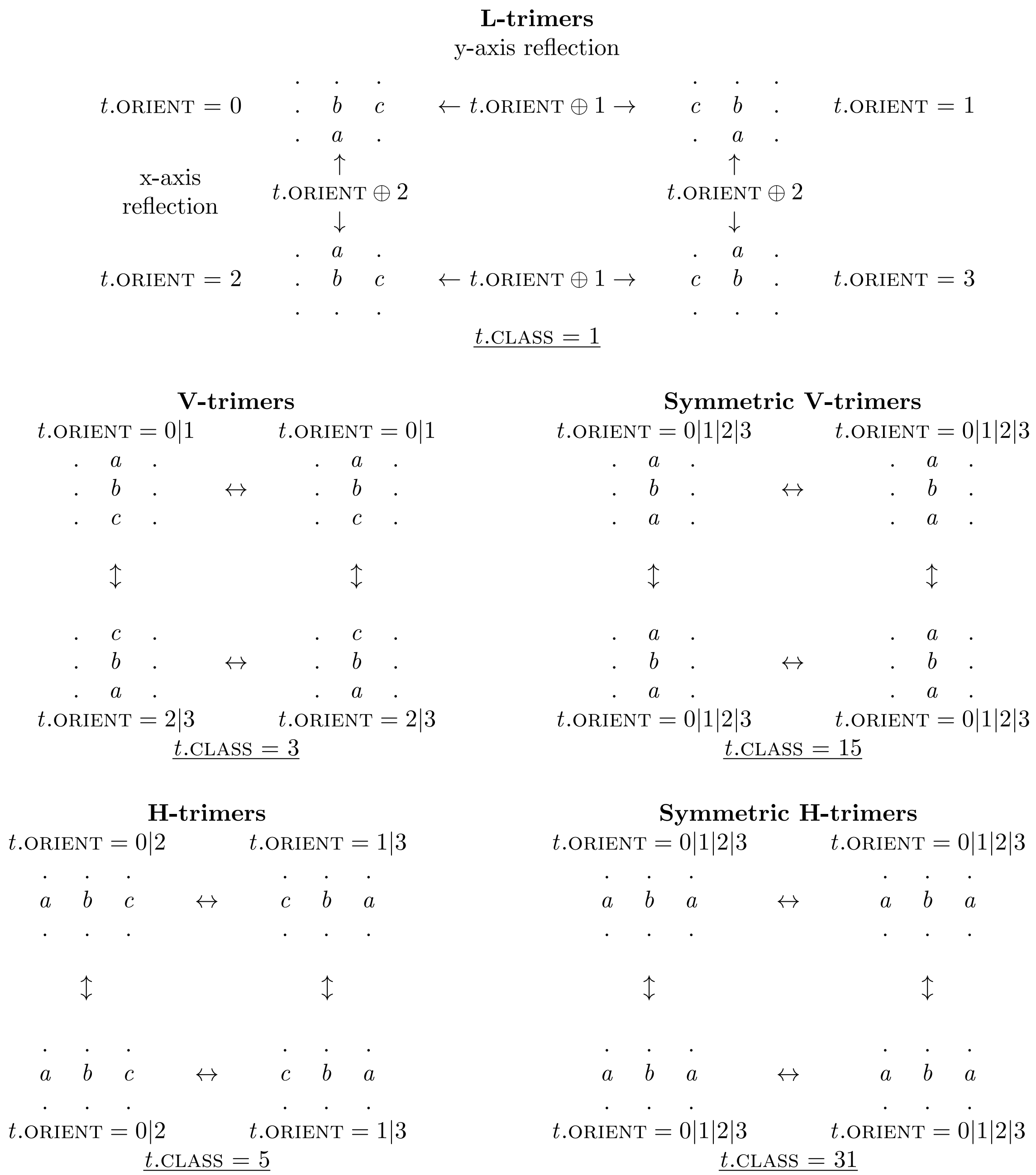

Figure 4: The L- (top), V- (middle), and H-trimer (bottom) orientations. Each L-trimer has a unique orientation due to their asymmetry in both axes. V-trimers are symmetric in the y-axis, resulting in an orientation equivalency of 0 with 1; and 2 with 3. Similarly, H-trimers are symmetric in the x-axis, resulting in an orientation equivalency of 0 with 2; and 1 with 3. Trimers with a t.CLASS of 15 or 31 are known as symmetric trimers because they are symmetric in both axes.

Algorithm 4: MAKE-QUERY-GRAPH returns a graph in which each edge denotes an overlap between two trimers.

MAKE-QUERY-GRAPH(Q)

1. *col-spans* and *row-spans* map unordered spans to lists of trimers where each list implies a clique in G.
2. For each trimer q in Q.TRIMERS and span s in q.SPANS, where s is converted to an unordered span.
   (a) If s is a row span, set *row-spans*$[s] \leftarrow$ *row-spans*$[s] \cup q$.
   Otherwise, set *col-spans*$[s] \leftarrow$ *col-spans*$[s] \cup q$.
3. For each span s in *col-spans*, add an edge $(t_{\text{src}}, t_{\text{des}})$ to G for each pair of trimers in *col-spans*$[s]$.
4. For each span s in *row-spans*, add an edge $(t_{\text{src}}, t_{\text{des}})$ to G for each pair of trimers in *row-spans*$[s]$.
5. Return G.

Algorithm 5: BFS-Match.

BFS-Match$(G, S, q_{root}, t_{\text{src}})$

1. Set *matched* $\leftarrow$ True.
2. For each pair of adjacent trimers (q_{src}, q_{des}) in a breadth-first traversal of G beginning at q_{root}.
   (a) The orientation of t_{des} is
   $orient \leftarrow$ Rel-Orient $\oplus$ t_{src}.orient.
   (b) The overlapping span numbers between t_{src} and t_{des} are
   $(s_{src}, s_{des}) \leftarrow$ Overlap-Span-Nums$(q_{\text{src}}, q_{\text{des}})$.
   (c) The overlap type between t_{src} and t_{des} is
   ol-type $\leftarrow$ Overlap-Type$(q_{\text{SRC}},\ q_{\text{DES}})$.
   (d) The span in t_{src} that overlaps with t_{des} is
   $span_{src} \leftarrow S[t_{\text{src}}][s_{src}]$.
   (e) Determine the following compound key values for t_{des}

$span_{des}$	:	the span in t_{des} that overlaps with t_{src}
coord	:	the row or column coordinate of t_{des}
I	:	the row or column span index in which to find t_{des}

   where each value is calculated as

   $$(span_{des}, pos, I) = \begin{cases} (span_{src}, t_{\text{src}}.\text{ROW}, \mathcal{R}) & \text{if } ol\text{-}type = \text{Bridge} \\ (span^{-1}_{src}, t_{\text{src}}.\text{ROW}, \mathcal{R}) & \text{if } ol\text{-}type = \text{Opp-Bridge} \\ (span_{src}, t_{\text{src}}.\text{COL}, \mathcal{C}) & \text{if } ol\text{-}type = \text{Peptide} \\ (span^{-1}_{src}, t_{\text{src}}.\text{COL}, \mathcal{C}) & \text{otherwise.} \end{cases}$$

   (f) The span index compound key for t_{des} is
   $key \leftarrow (c.\text{SHEET-ID}, q_{\text{des}}.\text{ID}, q_{\text{des}}.\text{EQUIV-ORIENTS}, coord, span_{des})$.
   (g) Set $t_{\text{des}} \leftarrow I[key]$.
   (h) If $t_{\text{des}} = \emptyset$, set *matched* $\leftarrow$ False and break out of the loop.
   Otherwise, set t_{des}.orient $\leftarrow$ *orient*.
   (i) If $s_{des} = 1$, set $S[t_{\text{des}}][2] \leftarrow t_{\text{des}}.\text{SPAN2}$.
   Otherwise, set $S[t_{\text{des}}][1] \leftarrow t_{\text{des}}.\text{SPAN1}$.
3. Return *matched*.

Algorithm 6: Verify-Candidate.

Verify-Candidate$(G, S, q_{root}, t_{\text{src}})$

1. Set *matched* $\leftarrow$ True.
2. For each t_{src} in c.trimers where t_{src}.id $= q_{root}$.id.
   (a) The span table S maps a trimer and a span number to a span.
   Set $(S[t_{\text{src}}][1], S[t_{\text{src}}][2]) \leftarrow (t_{\text{src}}.\text{SPAN}1, t_{\text{src}}.\text{SPAN}2)$.
   (b) For each combination of span numbers in S, swap $S[t_{\text{src}}][1]$ with $S[t_{\text{src}}][2]$ after the first iteration if t_{src} is a palindromic trimer.
   This step is required because the span numbers for symmetric trimers are not well-defined, so the only way to obtain the "correct" span number assignments is to examine both combinations.
      i. Set *matched* $\leftarrow$ BFS-Match$(G, S, c, q_{root}, t_{\text{src}})$.
      ii. If *matched* $=$ False, break out of the loop.
      iii. If t_{src} is an aysmmetric trimer, break out of this loop after the first iteration.
      This occurs because an asymmetric trimer has only one possible span number combination.
   (c) If *matched* $=$ True, set $C_3 \leftarrow C_3 \cup c$ and Return True.
3. Return False.

Algorithm 7: Verify.

Verify(Q, C_2)

1. Build the overlap graph
   $G \leftarrow$ Make-Query-Graph(Q).
2. Set q_{root} to be a trimer in Q.trimers where q_{root}.id is the least frequently occurring trimer ID in Q.trimer-ids such that

$$q_{root}.\text{CLASS} = \begin{cases} 1 & \text{if } Q \text{ has L-trimers} \\ 3 \text{ or } 5 & \text{if } Q \text{ has asymmetric trimers} \\ 15 \text{ or } 31 & \text{otherwise.} \end{cases}$$

3. Set $C_3 \leftarrow \{c \in C_2 : \text{Verify-Candidate}\,(G, c, q_{root})\}$
4. Return C_3

Table 1: Dataset query counts, the number of queries generated for each query size.

		**Query size** (edges)								
		**2**	**3**	**4**	**5**	**6**	**7**	**8**	**9**	**10**
**Dataset size**	**1000**	999	827	965	824	722	798	717	675	678
	**2000**	1999	1678	1919	1675	1464	1611	1450	1348	1372
	**4000**	3999	3337	3853	3329	2957	3196	2930	2707	2793
	**8000**	7998	6725	7727	6718	6005	6461	5969	5513	5679
	**16000**	15994	13490	15376	13461	11961	12936	11886	10990	11321

Table 2: We used an existing amino acid grouping scheme [1] and considered the *non-polar* and *aromatic* residues to be hydrophobic ("h") and others hydrophilic ("p").

**Group**	**Amino acids**
non-polar	G, A, V, L, I, P, M
polar (neutral)	C, N, Q, S, T
charged	E, D, K, R, H
aromatic	F, Y, W

Algorithm 8: Helper procedures required for candidate verification.

REL-ORIENT($t_{\mathrm{src}}, t_{\mathrm{des}}$) $\rightarrow$ t_{src}.ORIENT $\oplus$ t_{des}.ORIENT

OVERLAPS($t_{\mathrm{src}}, t_{\mathrm{des}}$) $\rightarrow$ True if t_{src} and t_{des} have a span that overlaps, False otherwise.

OVERLAP-TYPE($t_{\mathrm{src}}, t_{\mathrm{des}}$)

1. Set ($span_{src}, span_{des}$) $\leftarrow$ OVERLAP-SPANS($t_{\mathrm{src}}, t_{\mathrm{des}}$).
2. If $span_{src}$ is a row span and $span_{src} = span_{des}$, set *type* $\leftarrow$ Bridge. Otherwise, set *type* $\leftarrow$ Opp-Bridge.
3. If $span_{src}$ is a column span and $span_{src} = span_{des}$, set *type* $\leftarrow$ Peptide. Otherwise, set *type* $\leftarrow$ Opp-Peptide.
4. If OVERLAPS($t_{\mathrm{src}}, t_{\mathrm{des}}$) = False, set *type* $\leftarrow$ No-Overlap.
5. Return *type*.

OVERLAP-SPAN-NUMS($t_{\mathrm{src}}, t_{\mathrm{des}}$) $\rightarrow$ a tuple (s_{src}, s_{des}), in which s_{src} is the span number of t_{src} that overlaps with t_{des} and s_{des} is the span number of t_{des} that overlaps with t_{src}.

References

[1] A. Kessel and N. Ben-Tal, *Introduction to proteins: structure, function, and motion.* CRC Press, 2011.

[2] J. J. Havranek, "Specificity in computational protein design," *J Biol Chem*, vol. 285, pp. 31095–31099, 2010.

[3] Y. Liu and B. Kuhlman, "RosettaDesign server for protein design," *Nucleic Acids Res*, vol. 34, pp. W235–W238, 2006.
